# Improving *in vitro* screening compounds anti-*Trypanosoma cruzi* by GFP-expressing parasites

**DOI:** 10.1590/0074-02760230223

**Published:** 2024-05-06

**Authors:** Cleyson Mathias Morais Delvoss, Alexandre Haruo Inoue, Rosiane Valeriano da Silva, Stênio Perdigão Fragoso, Iriane Eger

**Affiliations:** 1 Universidade Estadual de Ponta Grossa, Laboratório de Biologia Celular e Protozoologia, Ponta Grossa, PR, Brasil; 2 Fundação Oswaldo Cruz-Fiocruz, Instituto Carlos Chagas, Laboratório de Pesquisa em Apicomplexa, Curitiba, PR, Brasil; 3 Fundação Oswaldo Cruz-Fiocruz, Instituto Carlos Chagas, Laboratório de Biologia Molecular e Sistêmica de Tripanossomatídeos, Curitiba, PR, Brasil

**Keywords:** trypanocidal activity, Trypanosoma cruzi, GFP, drug screening

## Abstract

**BACKGROUND:**

Conventional microscopic counting is a widely utilised method for evaluating the trypanocidal effects of drugs on intracellular amastigotes. This is a low-cost approach, but it is time-consuming and reliant on the expertise of the microscopist. So, there is a pressing need for developing technologies to enhance the efficiency of low-cost anti-*Trypanosoma cruzi* drug screening.

**OBJECTIVES:**

In our laboratory, we aimed to expedite the screening of anti-*T. cruzi* drugs by implementing a fluorescent method that correlates emitted fluorescence from green fluorescent protein (GFP)-expressing *T. cruzi* (Tc-GFP) with cellular viability.

**METHODS:**

Epimastigotes (Y strain) were transfected with the pROCKGFPNeo plasmid, resulting in robust and sustained GFP expression across epimastigotes, trypomastigotes, and intracellular amastigotes. Tc-GFP epimastigotes and intracellular amastigotes were exposed to a serial dilution of benznidazole (Bz). Cell viability was assessed through a combination of microscopic counting, MTT, and fluorimetry.

**FINDINGS:**

The fluorescence data indicated an underestimation of the activity of Bz against epimastigotes (IC_50_ 75 µM x 14 µM). Conversely, for intracellular GFP-amastigotes, both fluorimetry and microscopy yielded identical IC_50_ values. Factors influencing the fluorimetry approach are discussed.

**MAIN CONCLUSIONS:**

Our proposed fluorometric assessment is effective and can serve as a viable substitute for the time-consuming microscopic counting of intracellular amastigotes.

American trypanosomiasis, also known as Chagas disease (CD), is a zoonotic illness caused by the protozoan *Trypanosoma cruzi*. Endemic to the Americas and trivialised by society due to its predominant impact on poor and vulnerable populations,^1^ this parasitic infection is classified as a neglected disease by the World Health Organization (WHO).^2^ The primary transmission mechanism of *T. cruzi* is through the vector pathway, but alternative routes of transmission include blood transfusion, organ transplantation, laboratory accidents, congenital, and oral infections. Oral transmission is particularly noteworthy in the medical scenario of CD due to its characteristic outbreak nature, clinical severity, and higher parasite inoculum compared to the classic vector transmission.^3^ These transmission mechanisms, combined with human migration between the Americas and other regions, have elevated CD to a global health concern.^4^

While blood donor screening for CD and vector control campaigns have been implemented in certain countries, successfully reducing the number of cases, these measures have not been sufficient to eradicate the disease^3^ owing to the wildlife cycle of *T. cruzi.* ^2^ Despite vector control success in specific regions, this is not the case for many CD-endemic countries, such as Bolivia, where transmission of *T. cruzi* by the vector route persists as a significant health challenge despite efforts to control the vectors.^3^ ^,^ ^5^

Currently, CD is treated with two drugs, benznidazole (Bz) and nifurtimox (Nfx), developed over 50 years ago.^6^ ^,^ ^7^ These drugs are primarily employed for treating acute cases, with limited efficacy in chronic cases, and their use in this stage lacks consensus.^6^ Additionally, both compounds induce severe side effects.^8^ ^,^ ^9^

Consequently, there is an urgent demand for a pharmacological treatment applicable to both clinical stages of CD. While some pharmaceutical industries have been willing to discover new trypanocidal compounds,^7^ the pursuit of novel anti-*T. cruzi* drugs primarily depends on initiatives by public research institutions, which face limited funding for CD research and are unable to acquire equipment for sophisticated screening.^2^ ^,^ ^7^ Initial screening of anti-*T. cruzi* compounds usually involves *T. cruzi* epimastigote forms and colorimetric methods like MTT or microscope counting.^10^ However, since epimastigotes (insect forms) are not found in mammalian hosts, using them for drug screening might yield results not applicable to amastigote and trypomastigote forms, present in mammalian hosts. Amastigotes are the most clinically relevant targets for anti-*T. cruzi* drug action in humans, with their evaluation typically relying on microscopic counting of the parasites within cells throughout treatment.^11^ Despite being reproducible, this method is time-consuming and dependent on the microscopist experience, hindering routine or simultaneous screening of numerous compounds.

Some approaches have been developed to overcome these drugs screening drawbacks, including the use of *T. cruzi* strains expressing recombinant proteins, such as the β-galactosidase or green fluorescent protein (GFP) and its variants. β-galactosidase catalyses a colorimetric reaction, and its optical density determined by spectrophotometry is directly proportional to the number of viable parasites in the sample.^12^ ^,^ ^13^ However, this method has some limitations such as irregular expression levels of the heterologous gene and high maintenance costs, which make it unsuitable for routine use. An alternative is measuring the fluorescence emitted by GFP-expressing *T. cruzi*, as fluorescence intensity correlates with the number and viability of parasites in a sample. Unlike *β*-galactosidase assays, fluorescence detection is cost-effective and can be observed under a fluorescence microscope or read on a fluorimeter and flow cytometer. GFP has been employed in trypanocidal compound screening, offering a semi-automated method that expedites testing.^14^ ^,^ ^15^ ^,^ ^16^ However, these assays were conducted with Dm28c clone^14^ - which does not represent the heterogeneity of a natural *T. cruzi* strain - or with YBM,^15^ or K98^16^ - human strains, which are not widely used for drugs screening purposes.

In this study, we detail the development of a *T. cruzi* Y strain engineered to express the GFP and assess its utility in anti-*T. cruzi* drug screening, specifically through Bz testing. The Y strain is acknowledged for its moderate susceptibility to Bz^17^ and it is extensively employed in assays for screening trypanocidal compounds. Additionally, the adoption of a reference strain facilitates the comparison of results across distinct studies.

## MATERIALS AND METHODS

*Parasites* - Epimastigote forms of *T. cruzi* Y strain^18^ were maintained at 27ºC by two passages weekly in liver infusion tryptose (LIT) medium supplemented with 10% heat-inactivated foetal bovine serum (FBS). Axenic trypomastigotes were obtained from stationary-phase culture in LIT medium and used for infection of Vero E6 cells (ATCC^®^ Number: CRL-1586™) cultured in DMEM + 10% FBS. Cell-culture-derived trypomastigotes were harvested and used for maintenance of the intracellular cycle.

*Transfection of T. cruzi* - Transfection of *T. cruzi* Y strain was carried out as previously described,^14^ using the linearised pROCKGFPNeo plasmid,^19^ ^,^ ^20^ an integrative vector for stable expression of GFP in *T. cruzi*.^20^ Briefly, epimastigotes were cultured in LIT + 10% FBS medium to a density of 2x10^7^ parasites/mL, washed twice in phosphate buffered saline (PBS), centrifuged at 4,000 x *g* for 5 min and resuspended in 0.4 mL of electroporation buffer (140 mM NaCl, 25 mM HEPES pH 7.5, 0.74 mM Na_3_HPO_4_) at a concentration of 1x10^8^ parasites/mL. The suspension was transferred to a 0.2 cm gap cuvette and homogenised with 10 µg of the pROCKGFPNeo plasmid. After 10 min on ice, the mixture was subjected to two pulses of 450 V and 500 µF in the Gene Pulser Xcell electroporator (Bio-Rad, Hercules, USA). The electroporated cells were kept in ice, for 5 min and transferred to a culture flask containing 10 mL of LIT + 10% FBS. After 24 h, 500 µg/mL of G418 antibiotic (Sigma, St. Louis, MO, USA) was added, and the parasites were maintained at 27ºC. Tc-GFP subpopulations with high expression were sorted by BD FACSAria™ II cell sorter at the flow cytometry facility at the Carlos Chagas Institute/FIOCRUZ-PR (PDTIS/Instituto Carlos Chagas - Fiocruz Paraná, Brazil). Parasites with high and homogeneous expression were pooled and Tc-GFP cultures were maintained in LIT + 10% FBS with 100 µg/mL G418.

*Immunofluorescence microscopy* - Tc-GFP epimastigotes were fixed with 4% paraformaldehyde for 30 min. Then, the parasites were resuspended in PBS + 3% BSA (Serum Bovine Albumin) and adhered to a coverslip for 1 h. For intracellular amastigotes, Tc-GFP-infected Vero E6 cells were seeded on circular glass coverslips distributed in 24-well plates with DMEM + 10% FBS, as previously described.^21^

The parasites were permeabilised with 0.1% Triton X-100 in PBS for 5 min and incubated with murine anti-GFP mAb (1:50) in PBS + 3% BSA for 1 h at room temperature. Then, the samples were incubated with Alexafluor 488-conjugated anti-mouse IgG antibody (1:1000) in PBS + 3% BSA for 1 h at room temperature, followed by incubation with DAPI (4’, 6’-diamino-2-phenyl-indole) for 5 min and then mounted with Fluoromount (Sigma). Samples were observed on a fluorescence microscope (Zeiss Imager A2).

*Comparative growth curve of epimastigotes* - Wild-type epimastigotes (TcWT) and Tc-GFP were maintained in LIT + 10% FBS medium at 27ºC with an initial inoculum of 5x10^6^ parasites/mL. The number of parasites was determined daily by counting in a Neubauer chamber under an optical microscope along eight days.

*Comparative Bz IC*_*50*_ - Epimastigotes (10^7^ parasites/mL) from TcWT and Tc-GFP were seeded in 96-well plates containing PBS + 5.4% glucose (PSG) + 10% FBS. They were incubated at 27ºC in absence or presence of 5 to 25 µM of Bz. After 48 h survival parasites were evaluated in the same sample by both colorimetric MTT assay^22^ and microscopic counting in a Neubauer chamber.

*Selection of an appropriate medium for fluorimetric assays* - Aiming to find a suitable autofluorescence-free medium for fluorometric assays, we tested LIT, Schneider, DMEM, PSG (PBS + 5.4% glucose), PSG + 10% FBS, and PSG + 20% FBS. The assays were carried out in a fluorimeter plate at an excitation/emission wavelength of 480/512 nm. Additionally, a viability test for *T. cruzi* in the selected medium was conducted.

*Epimastigote growth inhibition assay* - To investigate the utility of fluorescent Tc-GFP parasites in drug screening, we examined whether the fluorescence intensity correlated with the density of viable epimastigotes. Tc-GFP epimastigotes were distributed in a 96-well dark plate (Greiner Bio-One BR, SP, Brazil) at various cell densities (ranging from 0.05 to 50x10^6^ parasites/mL) in PBS. Fluorescence intensity was promptly measured using a plate fluorimeter (Synergy H1 Hybrid Reader, Biotek) with an excitation/emission wavelength of 480/512 nm. Subsequently, following the fluorometric assessment, the parasites were transferred to a clear plate and subjected to an MTT viability assay. The values obtained through fluorimetry were compared to those derived from viability analysis using MTT.

For drug testing, TcWT and Tc-GFP (10x10^6^ parasites/mL) were cultured in 96-well plates in PSG + 10% FBS and exposed to concentrations ranging from 0 to 100 µM of Bz. After 48 h incubation at 27ºC, trypanocidal activity was assessed through fluorescence, as described previously, both before and after plate centrifugation (2,000 x *g*, 7 min). Following centrifugation, both WT and GFP cultures were further evaluated through microscopic counting using a Neubauer chamber and MTT assay. Samples were collected for morphological analysis using a fluorescence microscope (Zeiss Imager A2).

*Intracellular amastigotes growth inhibition assay* - Previously, we evaluated the correlation between fluorescence intensity and viable intracellular amastigotes. Vero E6 cells were seeded in 96-well light and dark plates (15x10^3^ cells/well) in DMEM + 10% FBS medium and immediately exposed to varying Tc-GFP trypomastigote inocula (1, 5 and 10 parasites/cells) and maintained at 37ºC and 5% CO_2_. After 12 h incubation, non-internalised parasites were removed by washing with PBS and the plates were further incubated under the same conditions described above. Following 48 h of infection, the plates were washed twice with PBS. The dark plate was used to fluorimetric assay. The light plate was subjected to staining using May-Grünwald method. The intracellular amastigotes were quantified through manual microscopic counting using an inverted optical microscope (Olympus bx41).

For trypanocidal compound testing, Vero E6 cells were seeded on light and dark plates and infected as described above. After 24 h of incubation, the infected cells were exposed to 0 to 25 µM of Bz in DMEM + 10% FBS. After 24 h of incubation, the dark plate was subjected to the fluorimeter reader and the light plate was stained by the May-Grünwald method for determining the parasite load.

*Statistical analysis* - All statistical analyses were performed using variance analysis (ANOVA 2-way) using the Graph Prism Instat 7^®^. The p < 0.05 value was considered significant in all tests. IC_50_ values were obtained using the CompuSyn program.

## RESULTS

**Fig. 1:**
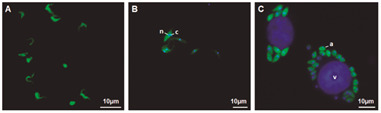
all stages of *Trypanosoma cruzi* transfected with pROCKGFPNeo express green fluorescent protein (GFP) *in vitro*. GFP expression was confirmed in trypomastigotes (A), epimastigotes (B), and intracellular amastigotes (C), in fresh samples (A), and fixed samples by immunofluorescence (B, C). Epimastigotes (B) and intracellular amastigotes (C), were incubated with DAPI (blue) and anti-GFP monoclonal antibody (green). n = nucleus, k = kinetoplast, a = amastigotes, and v = vero cell nucleus.

*All forms of T. cruzi showed GFP in vitro expression* - Fluorescent epimastigotes became evident starting from the 4th day post transfection with the pROCKGFPNeo plasmid. Following cell sorting, the transfected epimastigotes exhibited high GFP expression (Fig. 1B).

Notably, elevated GFP expression was also consistently observed in cell-culture-derived trypomastigotes and intracellular amastigotes (Fig. 1A and 1C), as evidenced by epifluorescence in fresh samples and anti-GFP immunofluorescence, respectively.

**Fig. 2:**
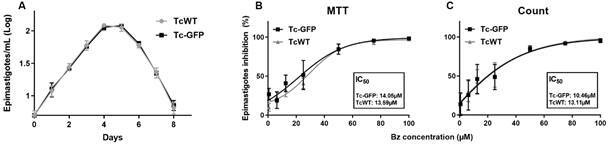
*Trypanosoma cruzi* epimastigotes transfected (Tc-GFP) and wild type (TcWT) have the same profile of growth (A) and susceptibility to benznidazole (Bz) (B, C). (A) Shows the growth curve of TcWT and Tc-GFP determined by Neubauer chamber counting. Both strains were seeded with initial inoculum of 5x10^6^ parasites/mL. The number of parasites was counted daily for eight days, and the results does not show statistical difference among the strains. (B) and (C) show *in vitro* assay of Bz effect on TcWT and Tc-GFP epimastigotes treated with different drug concentrations. In both cases, 10^7^ epimastigotes were seeded in 96-well plates using PSG [phosphate-buffered saline (PBS) + 5.4% glucose] + 10% foetal bovine serum (FBS) as a medium and incubated at 27ºC. Survival parasites were evaluated in the same sample by colorimetric MTT (B) and microscopic counting (C). Regardless of the method, the IC_50_ value was the same (p = 0.8863).

*GFP expression does not affect parasite growth and susceptibility to Bz* - We investigated whether GFP expression interfered with the growth and susceptibility of epimastigotes to Bz. As depicted in Fig. 2, both Tc-GFP and TcWT epimastigotes demonstrated comparable growth profiles and susceptibility to Bz. Furthermore, no alterations in infectivity and intracellular replication were observed throughout the entire duration of Tc-GFP maintenance in Vero cells (data not shown).

**Fig. 3:**
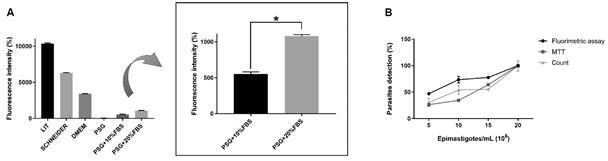
selection of an appropriate culture medium for anti-*Trypanosoma cruzi* screening compounds. (A) Liver infusion tryptose (LIT), a medium widely used for epimastigotes cultures, showed high autofluorescence. In detail, a comparison between PSG [phosphate-buffered saline (PBS) + 5.4% glucose] + 10% foetal bovine serum(FBS) and PSG + 20% FBS medium. (B) Shows data after growing epimastigotes in PSG + 10% FBS for 48 h to confirm their viability. Data were relativised considering the mean of the parasites in the largest inoculum as 100%.

*Selection of an appropriate medium for fluorimetric assays* - Autofluorescence from various media was detected at excitation and emission wavelengths proximate to those specified for GFP. Owing to its autofluorescence, LIT medium is unsuitable for drug screening via fluorimetric GFP assays, as this background could potentially lead to an underestimation of trypanocidal activity. Consequently, PSG + 10% FBS was chosen as the assay medium due to its ability to sustain parasite viability for 48 h and exhibit minimal autofluorescence, thereby mitigating fluorescence background interference (Fig. 3B).

**Fig. 4:**
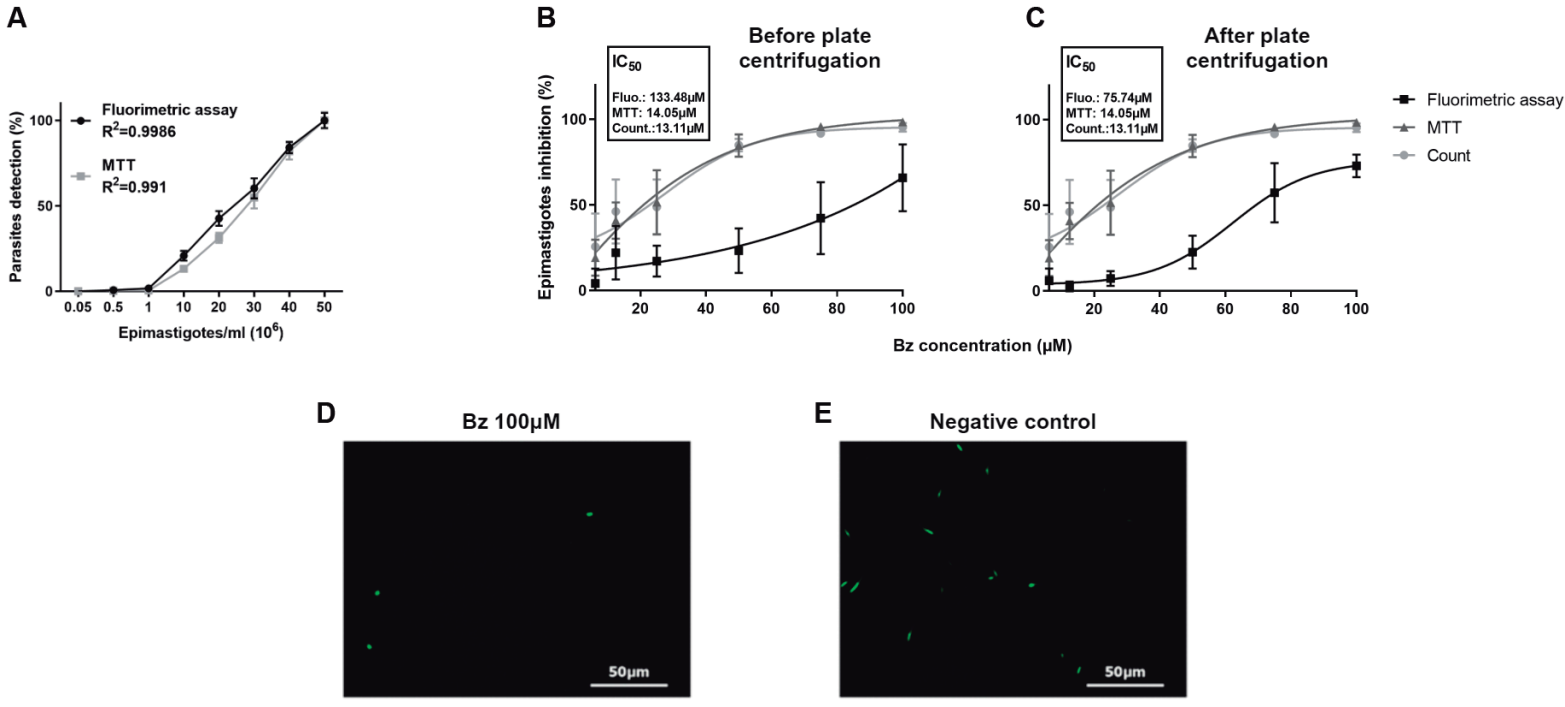
fluorimetry for drugs screening against *Trypanosoma cruzi* epimastigotes. In (A), different concentrations of epimastigotes were seeded in plates and subjected to MTT colorimetric assay and fluorimetric assay. Both methodologies show high detection correlation coefficient (R^2^) with parasites density. In (B) and (C), 10^7^ Tc-GFP epimastigotes were seeded in plates and incubated with different benznidazole (Bz) concentrations for 48 h. Epimastigotes viability was evaluated by Neubauer chamber counting, MTT and fluorimetry. Fluorometric analysis resulted in higher IC_50_ value (p = 0.0017), maybe by GFP retention in dead but not lysed parasites (D). In (E), the negative control with untreated viable parasites. Magnification: 400x.

*Fluorimetry for drug screening against epimastigotes* - We confirmed the direct correlation between fluorescence intensity and cell density of epimastigotes. The results demonstrated a highly significant correlation (R^2^ > 0.99) between the fluorescence signal of Tc-GFP parasites and the concentration of viable parasites when diluted in fresh PBS (Fig. 4A). This finding indicates that GFP expression reliably estimates parasite concentration in a sample, with a minimum detection limit of 0.5x10^6^ parasites/mL in 200 µL. This sensitivity is slightly superior to the MTT assay, which detects parasites at concentrations higher than 1x10^6^ parasites/mL in 200 µL. However, this correlation dissipated when Tc-GFP epimastigotes were exposed to Bz for 48 h. Fluorimetry exhibited higher IC_50_ values than MTT and microscopic counting in the same samples (Fig. 4B), thereby underestimating the effect of Bz. Notably, this background was not attributed to autofluorescence caused by Bz (data not shown). We hypothesised that the background might result from lysis of epimastigotes and residual detection of GFP released into the supernatant [Supplementary data (Figure A)]. Despite centrifugation effectively removing soluble GFP background [Supplementary data (Figure B-C)], the fluorescence signal remained higher than the number of viable epimastigotes even after washing the plate by centrifugation (Fig. 4C). Subsequent investigation suggested that the fluorescence background was due to GFP retention in non-lysed, dead epimastigotes. Monitoring of the Bz-treated Tc-GFP epimastigotes through fluorescence microscopy indicated that the parasites were not lysed, and they were still fluorescents (Fig. 4D) even when exposed to a high concentration (100 µM) of Bz. Nevertheless, these parasites exhibited changes in morphology (round shape - Fig. 4D) and were confirmed as dead through microscopic counting and MTT (Fig. 4C). Consequently, our results imply that the fluorometric assay for epimastigotes is only applicable for screening lytic drugs and requires an additional centrifugation step to remove GFP from the supernatant before fluorimetry reading.

**Fig. 5:**
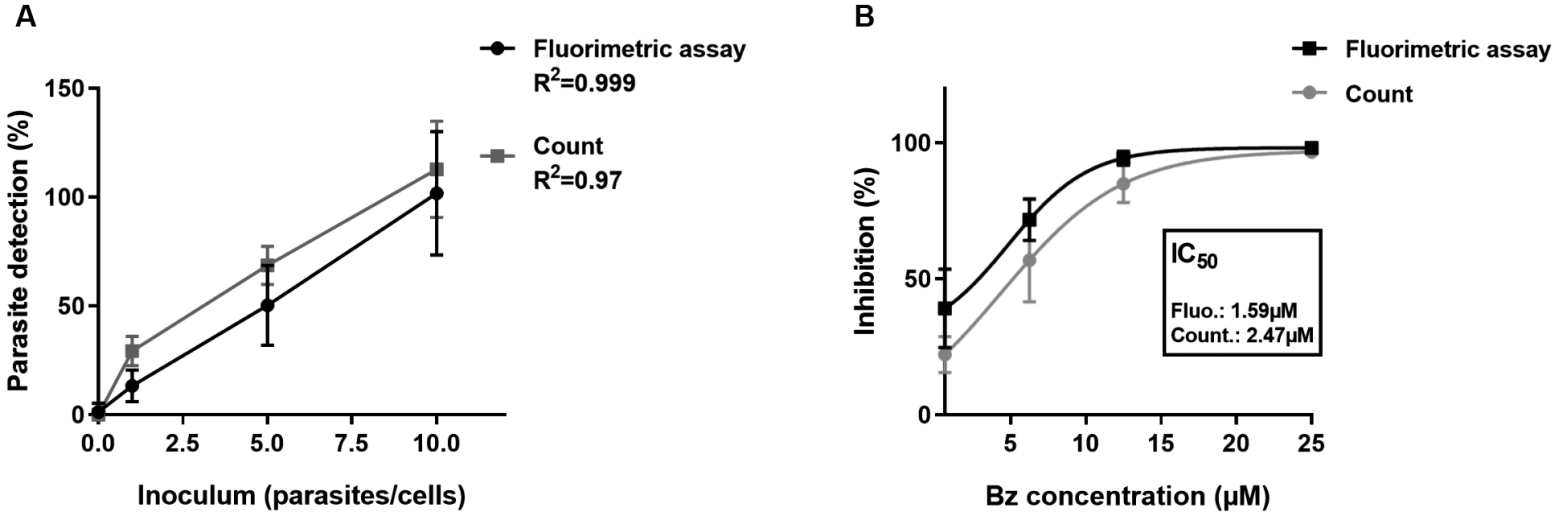
fluorimetry for screening drugs against intracellular amastigotes. In (A), vero E6 cells (15x10^3^ cells/well) were seeded in plates and exposed to different inoculum of Tc-GFP trypomastigotes. The intracellular amastigotes number was determinate by fluorescence assay and microscopic count 48 h after infection. Both methodologies have a high correlation index (R^2^ ≥ 0.97) between the initial inoculum of trypomastigotes and the intracellular amastigotes detection. There is no difference between the methods (p = 0.087). In (B), vero E6 cells are infected as described above and exposed to different benznidazole (Bz) concentration for 24 h. The intracellular amastigote number was determinate by fluorescence assay and microscopic count. Similar IC_50_ values were obtained in both methods (p = 0.064).

*Fluorimetry for screening drugs against intracellular amastigotes* - To evaluate whether fluorescence intensity correlated with intracellular amastigotes load, we infected Vero E6 cells with increasing GFP-trypomastigotes inoculum. As expected, after 48 h, the intracellular amastigotes load increased in accordance with the initial trypomastigotes inoculum. The infection rate was simultaneously evaluated by microscopic counting and fluorimetry. We verified that the fluorescence intensity signal of intracellular Tc-GFP amastigotes was directly proportional (R^2^ ≥ 0.97) to the parasite load estimated by microscopic counting (Fig. 5A). No fluorescence was detected in uninfected cell monolayers.

Subsequently, we treated infected Tc-GFP Vero E6 monolayers with a serial dilution of Bz and compared the drug effect using both fluorometric assay and microscopic counting. Regardless of the method employed, we obtained the consistent patterns of Bz susceptibility curves and similar IC_50_ values (p = 0.064) (Fig. 5B). These results suggest that a semi-automated fluorimetry method can effectively substitute the time-consuming technique of microscopic counting for drug screening against intracellular amastigotes of *T. cruzi*.

## DISCUSSION

Although numerous trypanocidal compounds are currently under evaluation from chronic Chagas disease,^7^ the drug screening process is challenging and time-consuming primarily due to the slow pace of the prevailing technique involving microscopic counting of residual intracellular parasites.^11^ To expedite the screening of new compounds, we propose a semi-automated method based on detecting fluorescence emitted by GFP expression in transfected parasites, offering a cost-effective, fast, and safe alternative. Given sustained GFP expression, the screening can encompass epimastigotes, intracellular amastigotes, and cell-culture-derived trypomastigotes. A comparable work was previously carried out using the *T. cruzi* clone Dm28c transfected with pBEX/GFP plasmid aimed to standardise a method for detecting trypanocidal compounds.^14^ However, given the high heterogeneity of *T. cruzi*,^23^ ^,^ ^24^ relying on a clone like Dm28c, might significantly limit the results.

Another study aimed to insert the gene for GFP expression in *T. cruzi* genome was carried out using the pROCKGFPNeo plasmid, transfected in the Tulahuén strain.^20^ In contrast to the pBEX/GFP plasmid, it inserts into the β-tubulin *loci*, contains a ribosomal promoter, a 5’ UTR sequence of TcP2β, sequences of glyceraldehyde 3-phosphate dehydrogenase glycosomal (gGAPDH) gene, and the phosphotransferase neomycin gene. Due to these characteristics, this plasmid exhibited constitutive expression of GFP in all evolutive stages of the parasite. The absence of a ribosomal promoter integrated with the plasmid would have led to expression conditioned by native promoters, inactivated by chromatin remodelling after differentiation into trypomastigotes.^25^ The presence of the 5’ UTR sequence of TcP2β allows for the addition of mini-exon via *trans*-splicing^20^ and the sequences of the gGAPDH gene ensure its constitutive expression and processing via polyadenylation, guaranteeing the stability of the transcribed mRNA. The neomycin phosphotransferase gene was included for resistance selection using the antibiotic G418.^26^

Here we employed the same plasmid^19^ ^,^ ^20^ mentioned above to transfect Y strain,^18^ known for this moderate resistance to Bz,^17^ ^,^ ^24^ making it an interesting and representative model for evaluating the trypanocidal effect of new compounds.

We observed GFP expression in all evolutive stages throughout the entire parasite, including the flagellum, although the levels of expression were occasionally heterogeneous (data not shown). The levels of GFP expression in all stages of the parasite remained homogeneous after G418 selection, as confirmed by direct fluorescence for the trypomastigote forms (Fig. 1A) or by indirect immunofluorescence for the epimastigote (Fig. 1B) and amastigote forms (Fig. 1C).

An essential criterion for replacing a wild-type strain with a transfected one is the maintenance of biological characteristics. In our experimental conditions, the growth curve of epimastigotes (Fig. 2A) and Bz susceptibility (Fig. 2B-C) were identical for TcWT and Tc-GFP. The next step was to confirm whether the emitted fluorescence correlated with live parasite density and to determine the ideal medium with no fluorescence background.

For epimastigotes, the fluorescence intensity was compared with the values sobtained by MTT colorimetric assay.^22^ Using PBS as the medium, the optical density and fluorescence were proportional to the concentration of parasites (Fig. 3A). However, PBS alone does not support the survival of epimastigotes during drug incubation, as tests are usually performed for 24 to 72 h. On the other hand, we detected autofluorescence of the conventional LIT medium at emission and excitation wavelengths like those described for GFP.^27^ This background affected the correlation between epimastigotes density and fluorescence signal (Fig. 3A). This effect was not caused by the spread of fluorescence between nearby wells, as dark plates absorb dispersed fluorescence and prevent it from spreading to adjacent wells. Therefore, due to the high autofluorescence, LIT medium is not appropriated for drug screening using fluorometric GFP assays, as this background may underestimate the trypanocidal activity.

Since epimastigotes are free-swimming cells, it is suitable to carry out the assays in a medium that can be directly read by the fluorimeter, avoiding a centrifugation step to replace the medium.

So, we replaced the LIT medium by PSG + 10% FBS for the epimastigotes assays (Fig. 3B). We also checked if GFP could remain in the supernatant, generating a detectable signal, even after parasites lysis, as this is one of the effects of trypanocidal drugs [Supplementary data (Figure)]. This idea is based on some GFP characteristics, such as (1) stability over a wide pH range (between 5.5 and 12); (2) fluorescence emitted even at high temperatures, close to 65ºC^28^ and; (3) the ability to remain fluorescent in the extracellular medium when GFP is secreted.^29^ Samples with lysed parasites showed lower fluorescence than the intact ones but were higher than the negative control - medium without parasites [Supplementary data (Figure A)], indicating the presence of soluble and detectable GFP in fluorimetry. This background was eliminated after we centrifugated the assay plates and replaced the medium with fresh PBS [Supplementary data (Figure B-C), demonstrating that residual GFP can be removed by centrifugation. Therefore, fluorescence of residual soluble GFP and LIT autofluorescence are limiting factors for anti-epimastigotes drug screening based on GFP fluorometric analysis. Besides, the autofluorescence of the compounds can interfere in fluorimetric assays, as most of the reagents have autofluorescence in a blue-green spectrum, which can cause interference in fluorescence readings that use such wavelengths.^30^ So, centrifugation step can be useful to remove both autofluorescent compounds and residual GFP from lysed epimastigotes.

After overcoming the two impediments to using fluorimetry as a method for trypanocidal compound assays in epimastigotes, we performed an activity test using Bz, and compared these results with Neubauer chamber counting and colorimetric MTT assay. Even when using a medium without autofluorescence and centrifuging the plate to remove residual fluorescence, the values obtained by fluorimetry were consistently higher than those obtained by the other methods, thus potentially underestimating the trypanocidal activity (Fig. 4C). Since Bz does not show autofluorescence at low concentrations (data not shown), we hypothesised that the detectable fluorescence signal might be due to dying parasites without lysis, containing residual GFP inside them (Fig. 4D). Bz exerts its trypanocidal effects through oxidative stress involving covalent modifications of macromolecules, causing damage to the parasite DNA, proteins and lipids. The metabolites of Bz can also affect the metabolism of the protozoan, enhancing its trypanocidal effect without acting in a lytic way.^31^ ^,^ ^32^

Together, our results suggest that drug screening against GFP-expressing *T. cruzi* epimastigotes should be carefully evaluated to avoid potential errors. Although epimastigotes (insect forms) are easily cultured in laboratory conditions, these forms are not found in the mammalian hosts. Drug screening using epimastigotes could yield results that might not apply to the amastigote and trypomastigote forms found in the infected mammalian host. Since amastigotes are the most relevant targets for chronic Chagas disease, we focused on improving drug screening by fluorometric assays using intracellular amastigotes expressing GFP. Firstly, we observed a high correlation (R > 0.9) between fluorescence intensity and intracellular parasites load (Fig. 5A). Furthermore, we also observed similar Bz IC_50_ values when evaluating trypanocidal activity using microscopic counting and fluorimetry in experiments were performed simultaneously under the same conditions (Fig. 5B). Unlike epimastigotes, we did not observe background fluorescence, even when some dead intracellular amastigotes remained, probably due to dead parasites and GFP degradation by the host cell,^33^ inhibiting the signal in fluorimetry.

In conclusion, our data suggest that screening of active drugs against epimastigotes-GFP should be carefully evaluated due to: (1) LIT medium autofluorescence; (2) residual GFP fluorescence in the supernatant of lysed epimastigotes; (3) autofluorescence exhibited by certain test compounds (these last two sentences demand a centrifugation step before fluorometric analysis); and (4) detectable fluoresce in round-death epimastigotes without lysis, containing residual GFP. In opposite, we showed that Tc-GFP fluorescence assays can effectively replace time-consuming microscopic counting in *T. cruzi* anti-amastigote assays. The method has proven to be fast, easily executed, toxicity-free and highly consistent with intracellular parasite load. It will facilitate large-scale screening of trypanocidal compounds. Moreover, this methodology allows the use of parasite in subsequent assays, such as microscopic analysis of cell morphology. It is worth noting the potential of this technique for *in vivo* testing,^14^ ^,^ ^15^ ^,^ ^16^ ^,^ ^34^ providing real-time imaging of the antiparasitic effect of chemical compounds using a non-invasive, safe, and efficient method.
