# Supplementary material for: Improving in vitro screening compounds anti-Trypanosoma cruzi by GFP-expressing parasites
Source: Mem Inst Oswaldo Cruz. 2024 May 6;119:e230223. doi: 10.1590/0074-02760230223 (PMC11075634; doi:10.1590/0074-02760230223)
Supplement: Supplementary file 1 [file 1678-8060-mioc-119-e230223-s.pdf]

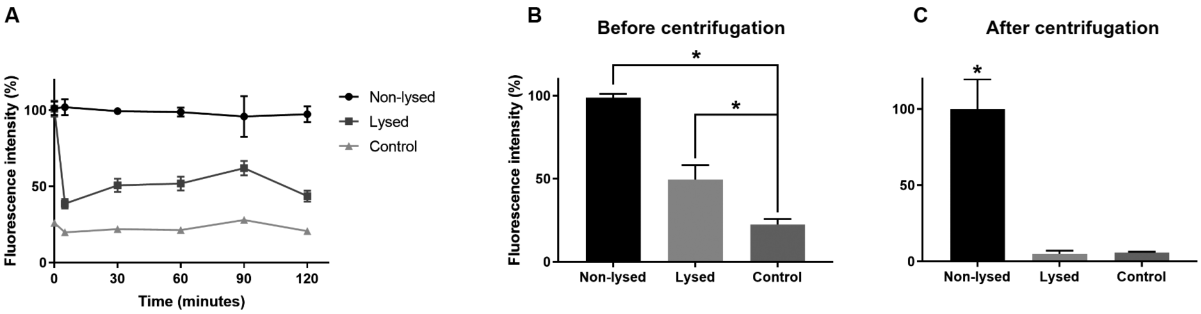

Centrifugation removes background of green fluorescent protein (GFP) in the supernatant from lised epimastigotes. Tc-GFP epimastigotes were collected from the cultures, washed 3x with phosphate buffered saline (PBS) and distributed in a 96 well dark plate at a concentration of 10^7 parasites/mL in PSG (PBS + 5.4% glucose) + 10% foetal bovine serum (FBS). After an initial reading fluorescence, 0.01M HCl + 10% sodium dodecyl sulphate (SDS) were added to smooth the parasite membrane. For negative control were used PSG + 10% FBS or PBS. (A) Shows the fluorescence kinetics data monitored every 30 min for 2 h. (B) GFP released to the supernatant from lysed epimastigotes was detected by fluorimetry, being significantly higher ($p < 0.0001$) in relation to the PSG + 10% FBS without parasites. (C) Plate centrifugation removes the residual fluorescence from lysed sample.

CC BY
